# Systemic Use of Arnica Montana for the Reduction of Postsurgical Sequels following Extraction of Impacted Mandibular 3^rd^ Molars: A Pilot Study

**DOI:** 10.1155/2020/6725175

**Published:** 2020-12-12

**Authors:** Hani Mawardi, Shaima Ghazalh, Ahmad Shehatah, Amnah Abdelwahid, Amin Aljohani, Osama Felemban, Soulafa Almazrooa, Lena Elbadawi, Hazem Shawky

**Affiliations:** ^1^Department of Oral Diagnostic Sciences, King AbdulAziz University, Faculty of Dentistry, Jeddah, Saudi Arabia; ^2^Ministry of Health, Madinah, Saudi Arabia; ^3^Ministry of Health, Taif, Saudi Arabia; ^4^Private Practice, Jeddah, Saudi Arabia; ^5^Department of Pediatric Dentistry, King AbdulAziz University, Faculty of Dentistry, Jeddah, Saudi Arabia; ^6^Department of Periodontics, King AbdulAziz University, Faculty of Dentistry, Jeddah, Saudi Arabia; ^7^Department Oral and Maxillofacial Surgery, Faculty of Oral and Dental Medicine, Cairo University, Cairo, Egypt; ^8^Department Oral and Maxillofacial Surgery, Al-Farabi Private College for Dentistry and Nursing, Jeddah, Saudi Arabia

## Abstract

**Background:**

Postsurgical sequels (PSS) are a group of complications commonly encountered following invasive dental surgical procedures such as bone grafting procedures, external sinus grafting, and 3^rd^ molar extractions. These include pain, intraoral and extraoral bruising, and edema. The aim of this study is to evaluate the clinical efficacy of arnica montana (AM) in the management of PSS following extraction of impacted mandibular 3^rd^ molars. The investigators null hypothesis includes no significant role of AM in reducing PSS following dental extraction.

**Materials and Methods:**

The investigators implemented a case-control pilot study enrolling twenty-three patients with impacted mandibular 3^rd^ molars. These patients were allocated to AM or control group. Baseline clinical measurements were collected and included: (1) length of the surgical procedure, (2) pain score, (3) maximum mouth opening, and (4) facial measurements to evaluate edema levels. Subjects in active group received systemic AM tablets following the manufacturer instructions. All study subjects were followed up on Days 2, 4, and 7. Data was analyzed for statistical significance.

**Results:**

A total of 30 impacted mandibular 3^rd^ molars were extracted, in which 22 completed with AM. There were 16 females, and the average age was 26 years. On Day 2, subjects in the AM group reported significantly lower VAS compared to control group (3.09 ± 2.22 versus 4.75 ± 1.28). In addition, bleeding, extraoral bruising, edema, and decrease in maximum mouth opening were significantly less reported in the AM group.

**Conclusions:**

This study describes the potential benefit of AM in reducing PSS following dental extractions.

## 1. Introduction

Postsurgical sequels (PSS) are a group of complications commonly encountered following invasive dental surgical procedures such as periodontal regenerative surgeries, maxillary sinus augmentation, and extraction of impacted 3^rd^ molars [1]. These include pain, extraoral bruising, trismus, edema, and few more [1, 2]. The degree and severity of PSS are often associated with several factors such as complexity of the surgical procedure, patient's underlying medical condition, age, procedure duration, and dental practitioner's experience [3]. The use of nonsteroidal anti-inflammatory medications (NSAID) and corticosteroids (CS) to prevent and manage PSS has been a common practice for decades with acceptable outcome and patient satisfaction [4–8]. Even with variability of outcome based on factors such as the severity of the surgery, patient's age, and surgical technique, special group of patients with chronic underlying medical conditions (e.g., hypertension, kidney disease, and diabetes mellitus) may not be eligible for such approach due to potential adverse events and toxicities affecting various body organs. As a result, other alternative pharmacological and homeopathic therapies with better safety profile should be considered [9, 10].

Several homeopathic remedies for management of PSS and other inflammatory conditions have been in use since the eighteenth century. Arnica Montana supplements (AM) is one, which has been introduced as an effective agent to reduce PSS and specifically edema [10]. It is an extract from a plant species which belong to the Asteraceae family comprising arnica montana, arnica fulgens, and arnica chamissonis commonly found in Central Europe and the Siberian mountains [11]. AM has been marketed in different formulation including topical application for skin and as a systemic preparations for oral consumption as well [9]. Emerging evidence from the medical literature has suggested for AM to replace common practices of prescribing NSAIDs and other available agents to reduce dental PSS with less potential toxicities [9]. In general, AM is considered safe for human consumption and has been long utilized with minimal reported adverse events [12]. As of today, the literature on AM application and efficacy in the dental field is still lacking.

The objective of this study is to evaluate the clinical efficacy of systemic AM in the management of PSS following extraction of impacted mandibular 3^rd^ molars. We believe this case-control study will be a great addition to the literature of PSS management in invasive dental procedures.

## 2. Methods

A human research ethical approval was obtained through Al-Farabi Private College, School of Dentistry, Jeddah, Saudi Arabia. The study included patients with impacted mandibular 3^rd^ molars indicated for extraction. Eligibility criteria for participation included (1) adult patients 18 years old or older; (2) impacted mandibular 3^rd^ molar with classes II–III surgical difficulty and depth level of B or C indicated for extraction based on Pell-Gregory classification (class II: the space between the second molar and the ramus of the mandible is less than the mesiodistal diameter of the third molar; class III: all or most of the third molar is in the ramus of the mandible; Depth B: the occlusal plane of the impacted tooth is between the occlusal plane and the cervical line of the second molar; and depth C: the impacted tooth is below the cervical line of the second molar); (3) no recent history of using NSAIDs or CS in the last 2 weeks; (4) no known underlying medical conditions which may affect the outcome of the study; and (5) no smoking history for at least the past 2 weeks prior to the surgery [13]. The study exclusion criteria included (1) patients with contraindication to AM therapy such as pregnancy and/or breastfeeding women; (2) active smoking of any type; and (3) known allergy history to AM.

**Figure 1:**
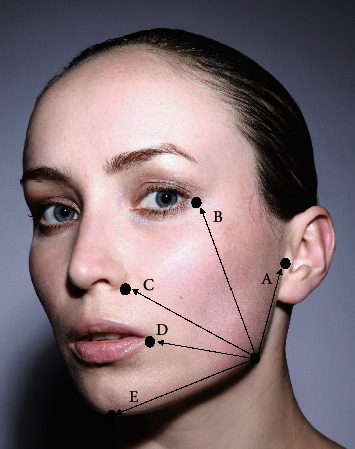
Facial landmarks and distances to angle of the mandible used to evaluate study subjects for facial edema at baseline and Day 7 (A = distance to tragus, B = distance to lateral canthus, C = distance to alar base, D = distance to lip commissure, and E = distance to pogonion).

The study aim and design were explained to all participants in detail prior to signing the consent form. Data on patient age, gender, medical history, and medications was collected for each subject. Baseline clinical measurements for all subjects included (1) classification of the impacted mandibular 3^rd^ molar; (2) length of the surgical procedure defined as the time between starting the 1^st^ surgical incision up until the last suture; (3) pain levels recorded on a visual analogue scale (VAS) of 10 cm with a score ranging from 0 (no pain) to 10 (the worst pain possible); (4) maximum mouth opening determined by measuring the interincisal distance; and (5) facial measurements to evaluate edema level at baseline was completed using facial anatomic landmarks as described previously by Neupert et al. [14]. Based on this protocol, the mandibular angle was used as a main reference point to measure the linear distances to the following facial landmarks: mandibular angle to tragus (A), to lateral canthus (B), to alar base (C), to lip commissure (D), and to pogonion (E) for each participant (Figure 1). The facial landmarks were identified using an indelible ink. All measurements were completed using a 3-0 silk suture following the face contour and documented in centimeters.

The extraction of all 3^rd^ molar procedures was performed by a single oral surgeon (HS) in order to limit potential differences in clinical skills and techniques which may impact the study outcome (Day 0). Extraction of mandibular 3^rd^ molars were completed under local anesthesia using 2% lidocaine with 1 : 100,000 epinephrine injected via inferior alveolar nerve (IAN) combined with buccal and lingual nerve block. A flap following Lotter design was raised using a 15C blade followed by guttering of the buccal and distal sides of the impacted tooth using an externally irrigated straight hand piece with surgical carbide bur (30,000 rpm) [15]. Teeth sectioning was completed as needed followed by tooth removal and curettage with smoothing of bone edges. Minimal modifications to the extraction steps took place for few cases as indicated which did not affect the study outcome. The extraction site was sutured with a 3-0 silk suture and hemostasis achieved. Ice pack was applied to the surgical side continuously for 20 minutes. Postsurgical instructions included antibiotics (amoxicillin 500 mg/3 times a day or clindamycin 300 mg/3 times a day for 5 days), paracetamol 500 mg, or paracetamol 500 mg/codeine, 8 mg/caffeine, and 30 mg (Solpadine®) every 6 hours for 3 days and then as needed until Day 7. Chlorhexidine gluconate antiseptic solution 0.12% was prescribed twice/day swish and spit starting Day 1 and for 7 days. No NSAIDs were prescribed after surgical procedure per study protocol.

Eligible patients were allocated to have the extraction of impacted mandibular 3^rd^ molar completed with or without AM. For cases with bilateral impaction of mandibular 3^rd^ molars, one side was extracted without AM first followed by extraction of the opposite side on AM with 2 weeks in between in order to avoid any latent effect on the control side. The protocol for study group included receiving AM tablets 30X (Hyland's Inc., Los Angeles, California) following the manufacturer instructions via dissolving each tablet under the tongue and then swallowing it per the following sequence: (1) 4 tablets 1 hour before the procedure (Day 0); (2) 4 tablets × 4 times/day starting 1 hour following extraction of mandibular 3^rd^ molar (Day 0; total of 16 tablets); (3) 4 tablets × 4 times/day on Day 1 (total of 16 tablets); (4) 4 tablets × 4 times/day on Day 2 (total of 16 tablets); and (5) 4 tablets × 4 times/day on Day 3 (total of 16 tablets).

On Days 2 and 4, participants were contacted by a study coinvestigator by phone to assess pain level (0 = lowest; 10 = highest). In addition, questions on the presence of active bleeding, skin bruising (ecchymosis), and limitation in mouth opening were asked using none/mild/moderate/severe scale. Patients were also asked about extraoral swelling (edema) using the following grading: 0 for no swelling, 1 for mild swelling, 2 for moderate swelling, and 3 for severe swelling.

All study subjects were followed up in the dental clinic on Day 7. During this visit, reevaluation of maximum mouth opening and facial edema was completed. In addition, healing progress of the surgical site in terms of bleeding, signs for infection or dry socket, and extraoral bruising was evaluated. Bleeding events were graded using WHO scale for oral bleeding defined as follows: Grade 1, total duration of all bleeding episodes in previous 24 hours is <30 minutes with petechiae of oral mucosa (mild); Grade 2, total duration of all episodes in previous 24 hours is >30 minutes (moderate); and Grade 3, any bleeding requiring RBC transfusion over routine transfusion needs (severe). In addition, clinical images were obtained at baseline and follow-up visits for comparison purposes.

Collected data were found to be nonnormally distributed. Continuous variables were analyzed using Mann–Whitney *U* test, while categorical variables were analyzed using Chi-Square test and Fisher Exact tests to compare the AM and control groups at the significance level of 0.05. SPSS Statistics for Windows®, Version 23.0 (Armonk, NY: IBM Corp) was used to analyze the data.

## 3. Results

There were 16 patients with unilateral and 7 patients with bilateral impacted mandibular 3^rd^ molars indicated for extraction. All 23 patients have completed the study, in which 16 patients (69.5%) were females and overall average age was 26 years (range 18–35). Study participants were asymptomatic at baseline and referred from the orthodontics service for extraction of impacted 3^rd^ molars. In general, all subjects were healthy without significant medical history and taking no medications. In addition, all subjects were not active smokers. Complete demographic data are summarized in Table 1.

**Figure 2:**
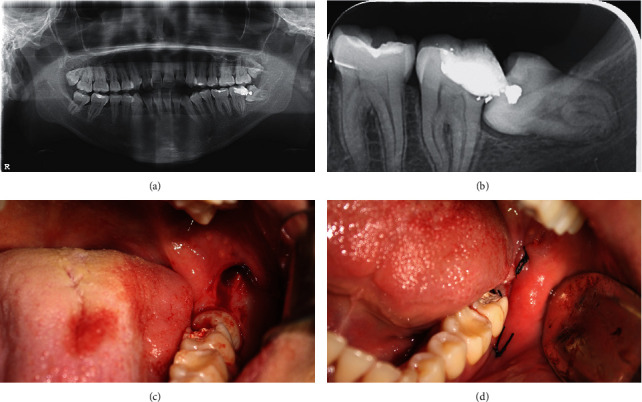
Surgical extraction of mandibular left 3^rd^ molar for Case 1. Presurgical evaluation included panoramic (a) and periapical (b) radiographic assessment in addition to clinical examination. The procedure was started by a pyramidal flap incision followed by mucoperiosteal flab elevation (c). Next, bone removal to expose the impacted molar was completed as needed followed by guttering buccally and distally. The site was sutured with simple interrupted sutures using 3-0 silk and hemostasis achieved (d).

**Figure 3:**
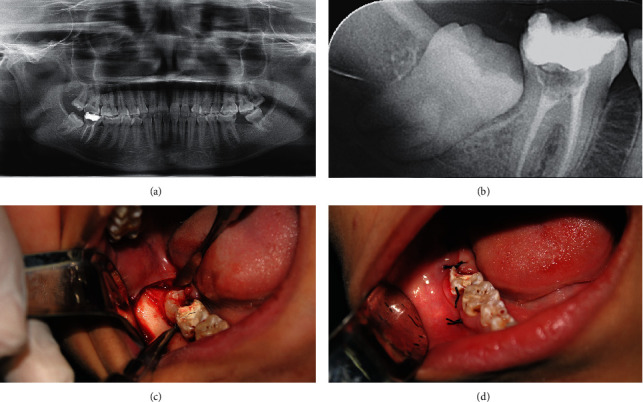
Surgical extraction of mandibular right 3^rd^ molar for Case 2. Presurgical evaluation included panoramic (a) and periapical (b) radiographic assessment in addition to clinical examination. The procedure was started by a pyramidal flap incision followed by mucoperiosteal flab elevation (c). Next, bone removal to expose the impacted molar was completed as needed followed by guttering buccally and distally. The site was sutured with simple interrupted sutures using 3-0 silk and hemostasis achieved (d).

Total of 30 mandibular 3^rd^ molars were extracted, in which 22 were in the AM group (Figures 2 and 3). No statistical difference was detected in terms of mandibular 3^rd^ molar depth (*p*=0.825) or class (*p*=1.00) between both AM and control groups. The mean extraction procedure duration was 37 minutes (range 30–45 minutes) for AM group and 39 minutes (range 32–43 minutes) for control group. On the day of the surgery, average maximum mouth opening for patients who received AM supplements was 4.79 ± 0.44 cm compared to 4.75 ± 1.28 cm for patients who did not receive it (*p*=0.872). In addition, all patients had pain VAS of 0 out of 10.

**Figure 4:**
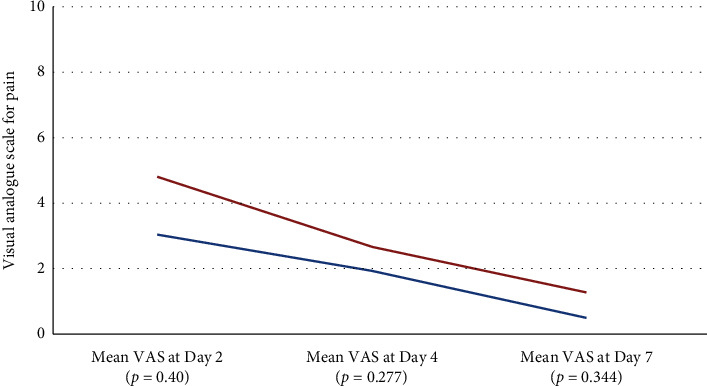
Reported VAS pain score on Days 2, 4, and 7.

Postsurgery follow-ups of study participants were completed via phone calls on Days 2 and 4 (Table 2). On Day 2, subjects in the AM group reported significantly lower pain VAS of 3.09 ± 2.22 compared to 4.75 ± 1.28 for control group (*p*=0.040). On Days 4 and 7, the AM group showed consistently lower mean pain VAS, but the differences were not statistically significant. On Day 4, the reported pain VAS was 1.86 ± 1.49 for AM group and 2.63 ± 1.60 for the control group (*p*=0.277). At the same time, pain VAS on Day 7 for AM group was 0.45 ± 1.26 and 1.25 ± 1.91 for the control group (*p*=0.344) (Figure 4). Bleeding was significantly less reported in the AM group compared to the control group (*p*=0.004) on Day 2. The majority of sites (72.7%) in the AM group had no bleeding reported, while the majority of sites in the control group (75.0%) reported Grade 1 bleeding. None of the patients in both groups reported any bleeding on Day 4.

In terms of extraoral swelling (edema), no statistically significant difference was found between the groups regarding the distribution of swelling severity on Day 2 (*p*=0.085). However, the swelling was significantly less severe in the AM group compared to the control group (*p*=0.027). In the AM group, 7 sites (31.8%) had no swelling, 12 sites (54.5%) had mild swelling, 2 sites (9.1%) had moderate swelling, and 1 site (4.5%) had severe swelling. For the control group, 1 site (12.5%) had no swelling, 2 sites (25%) had mild swelling, 1 site (12.5%) had moderate swelling, and 4 sites (50%) had severe swelling. On Day 2, extraoral bruising was significantly less severe in the AM group compared to the control group (*p*=0.029). Mild extraoral bruising was reported for 2 sites (9.1%) in the AM group compared to 4 sites (50%) in the control sites. By Day 4, no extraoral bruising was reported for 16 sites (72.2%) in the AM group; however, 3 sites (13.6%) had mild and 3 sites (13.6%) had moderate bruising. For the control group, 5 sites (62.5%) had no bruising, 1 site (12.5%) had mild, 1 site (12.5%) had moderate, and 1 site (12.5%) had severe bruising. The differences in extraoral bruising were not significant between both groups (*p*=0.526).

The distribution of the reported severity of limited mouth opening was significantly less severe on Day 2 among AM group compared to control group (*p*=0.016). In the AM group, no limitation in maximum moth opining was reported in 5 sites (22.7%). However, 12 sites (54.65%) had mild grade, 4 sites (18.2%) had moderate grade, and 1 site (4.5%) had severe limitation in mouth opening. Comparing these numbers to control group, 1 site (12.5%) had no limitation in mouth opening, 2 sites (25%) had mild grade, and 5 sites (62.5%) had moderate grade. Similar findings in terms of distribution of limited mouth opening severity were reported between both groups on Day 4. In the AM group, limitation in maximum moth opining was absent in 7 sites (31.8%); however, 11 sites (50%) had mild grade, 3 sites (13.6%) had moderate grade, and 1 site (4.5%) had severe limitation in mouth opening. Comparing these numbers to control group, 1 site (12.5%) had no limitation in mouth opening, 2 sites (25%) had mild grade, 3 sites (37.5%) had moderate grade, and 2 sites (25%) had severe grade.

On Day 7, all subjects had a scheduled follow-up visit in the dental clinic to assess healing following the extraction procedure. No difference in maximum mouth opening between AM and control group was noted (*p*=0.565). In addition, the average facial measurements for edema assessment (distances A, B, C, D, and E) between both groups were comparable with no statistical significance (Table 3). Extraoral bruising was also assessed on Day 7 (results are not included in tables). Mild extraoral bruising was reported for 4 sites (18.2%) in the AM group and 1 site (12.5%) for the control group. Only 2 sites (12.5%) in the control group had moderate bruising (*p*=0.075). No dry or infected sockets were detected in any of the surgical sites for both groups for the whole duration of the study. In addition, no major complication or toxicity related to AM use was noted.

## 4. Discussion

Surgical extraction of impacted 3^rd^ molar is a common procedure performed on regular basis in the dental office [1]. It is estimated that 5 million extraction procedures of 3^rd^ molars are conducted annually in the US, in which 11000 may experience PSS [16]. These complications may play a role in patient's decision to have a 3^rd^ molar extracted even in situations of clear indication. The list of potential PSS includes pain, bleeding, trismus, bruising, and most importantly extraoral edema. Although most of these secondary complications can be managed efficiently, PSS continue to pose a daily challenge to the dental practitioner due to limitation in armamentarium available to prevent or decrease its severity [1].

In the past years, multiple protocols have been proposed to manage PSS in impacted 3^rd^ molar extraction cases [17]. These include measures such as cold application following surgical procedure as well as modification to flap design and introduction of piezosurgery with limited effect [18–20]. The application of platelet-rich plasma was recently introduced to expedite surgical site healing and reduce the risk of PSS with mixed outcomes [21]. Several studies looked at the role of alternative natural materials in managing PSS associated with dental extraction. For instance, the effect of intrasocket application of Manuka honey on postoperative pain was tested in a randomized, split-mouth controlled study of 33 subjects [22]. The study reported reduction in VAS scores in the first and second days for subjects in the Manuka honey group. Another study reported reduction in postextraction bleeding of mandibular teeth when green tea-impregnated gauze was applied in a randomized controlled trial of 62 subjects [23]. Compared to AM, our data have demonstrated significant reduction in postoperative edema, bruising, and trismus with no significant toxicity.

Out of all, the use of pharmacological agents such as NSAIDs and CS in different formulations has demonstrated the most predictable outcomes [4, 6, 24]. However, the application of these agents is either contraindicated for some patients or associated with detrimental side effects advising for alternative options with minimal toxicities. AM has been used in the medical field for some time now with promising outcomes. It is a homeopathic agent available commercially, over the counter in different formulations (i.e., capsules and gel) and used in several countries including Europe and North America. In particular, AM has been introduced to manage edema, ecchymosis, and pain following surgical procedures. The current study report on the potential application of AM to prevent or manage PSS associated with extraction of impacted mandibular 3^rd^ molars.

As of today, almost all of the data available on AM is originating from the medical literature. A case series including 13 subjects who went through rhinoplasty surgery with osteotomies and received AM for 3 days was reported [25]. All patients had accelerated postoperative healing and decrease in bruising and ecchymosis. Out of all, a single patient experienced mild itching and rash which resolved during the study follow-up duration. A recent study reported a faster resolution of postoperative sore throat, dysphagia, aphonia, and hoarseness in 2 patients following laryngeal mask insertion [26]. Both patients were treated with 3 doses of AM and reported symptoms resolution within 36 hours. On the other hand, a randomized, double-blinded clinical trial was conducted and included 27 subjects to evaluate the effect of topical AM hydrogel pads on ecchymosis following upper blepharoplasty [27]. After 30 days, the study failed to demonstrate a statistical significance between both groups in terms of ecchymosis or healing period.

In the dental literature, a single study from the 1980s looked at the potential use of AM in dental surgeries. In this randomized double-blinded clinical trial, the effect of AM and metronidazole on PSS following extraction of mandibular 3^rd^ molars was evaluated in 118 patients [28]. Study participants were randomly assigned to three groups: Group 1 received metronidazole (400 mg twice daily); Group 2 received AM (200 mg twice daily); Group 3 received placebo tablets. Pain score, edema, and trismus parameters were used to compare the outcomes of AM and metronidazole. At the end of the study, metronidazole reduced the incidence of pain and edema and enhanced the healing process following surgical extraction compared to AM and placebo groups but had no effect on trismus. In addition, AM was less effective than the placebo in this clinical study. These results in general contradict our data, which could be contributed to factors such as difference in assessment methods, surgical technique used, and classification of impacted teeth extracted during the course of the study. Considering the limited available literature on AM in the dental field, its application may still benefit patients at risk of PSS based on the such as in cases of external sinus augmentation and major bone grafting procedures on a case-by-case basis. Therefore, the use of AM in the current study was justifiable based on the comparative, recent medical literature [9, 29, 30].

In order to better evaluate the effect of AM, several parameters were assessed at different time points in this study. On Day 2, patients in AM group had better pain control and milder PSS in terms of intraoral bleeding, extraoral bruising, and trismus compared to control group. In addition, extraoral edema was significantly less reported in the AM group compared to control. On Day 4, the difference in postoperative edema was more evident between both groups as 31.8% of AM sites had no swelling compared to 12.5% sites of control group. During the follow-up visit on Day 7, most of PSS have resolved completely for both groups as anticipated for similar cases in general. However, lower pain scores for AM sites were reported compared to control sites (0.45 ± 1.26 versus 1.25 ± 1.91).

As of today, limited measures are available to prevent or reduce PSS following surgical extraction of impacted 3^rd^ molars. Hyaluronic acid (HA) is linear polysaccharides of the extracellular matrix which can be found in various body tissues. Several studies investigated the effect of HA deposition in extraction sockets of impacted 3^rd^ molars and demonstrated decrease in postoperative pain [31]. However, no role in reducing other PSS was reported. A randomized, double-blind, crossover study was conducted to compare between the effect of etodolac, naproxen, and diclofenac as PSS prophylaxis for patients receiving surgical extraction of 3^rd^ molars [32]. A total of 42 patients were included in the study and allocated to either Group A of etodolac (200 mg), Group B of naproxen sodium (275 mg), or Group C of diclofenac potassium (50 mg) to be administered 1 hour before the procedure and continued for 3–5 days afterward. At the end of the study, diclofenac potassium was significantly more effective in reducing postsurgical edema compared to other groups.

The effect of CS either through injectable or systemic routes on PSS has been investigated extensively in the past. A split-mouth, randomized triple-blind controlled clinical trial was conducted to compare the effect of dexamethasone (8 mg/day) to diclofenac sodium (50 mg/day) and codeine (50 mg/day) on patients receiving extraction of bilateral mandibular 3^rd^ molars [33]. The study demonstrated dexamethasone to be the most efficient in controlling postoperative pain and edema. In a randomized clinical trial, intralesional and intravenous dexamethasone given 1 hour before procedure was effective and superior to oral dexamethasone in decreasing postoperative pain and edema following surgical extraction of 3^rd^ molars [7]. Combination of CS with other agents such as NSAIDs for synergistic effect has also been investigated and was superior to dexamethasone and NSAIDs alone in controlling postoperative edema and pain [34].

Based on our data, AM showed significant potential to decrease the degree of edema in addition to pain experience following surgical extraction of impacted mandibular 3^rd^ molars. Even with all patients received antibiotic therapy, none were prescribed NSAIDs to eliminate the confounding risk. One factor to consider when assessing AM effect is the purity of product and additive contents if any. The commercial supplements are not overseen by food and drug administration in most countries; significant variations in AM contents may exist among manufacturers potentially affecting the overall clinical outcome [35]. AM dosing is another factor to consider which varied between conducted studies in the literature and has to be investigated in future studies and assessed for bias [36]. The literature on AM safety profile is also lacking. However, several larger studies have investigated the effect of AM in managing PSS associated with different procedures and failed to report major complications in included subjects even in the setting of chronic underlying medical conditions such as diabetes, hypertension, and renal disease [9, 12]. Overall, the literature reported side effects were limited to mild itching and rash [25]. Hence, it may be reasonable to consider AM as a fairly safe product for human consumption. As of today, AM interaction with other medication is not clear and should be evaluated on a case by case basis.

The current study is aimed at highlighting the potential role of AM in dental setting, specifically in extraction of impacted mandibular molar. Based on our data, AM could be offered to selective group of patients with concerns over postoperative edema, bruising, and/or pain. It is fair to anticipate the same benefit with using AM in other invasive dental surgeries such as sinus augmentation and guided tissue regeneration. However, future studies needed better evidence-based application.

This study has several limitations. First, the enrollment of larger number of study and control sites may have allowed for better evaluation of the study outcome and more justification for clinical application in the daily practice. However, this study helps to shed more light on the potential benefit of AM in reducing PSS associated with impacted 3^rd^ molars extraction and help to support future studies to confirm these finding. Second, a single dose of AM was used in this cases series. Comparison between different manufactures' products and dosing would have given better understating on the best way to apply AM in the dental field. Third, assessment of postsurgical pain, bleeding, and edema was self-reported on Days 2 and 4 which may have been biased and underreported. In-clinic patient assessment on Days 2 and 4 would be considered in the future. Fourth, factors such as unequal number of enrolled subjects in relation to gender, race, variability of used postsurgical medications, and comparing amount of bone removed during the extraction may have affected our results.

## 5. Conclusion

Based on the current findings, AM seems to have a potential benefit in management of secondary complications following surgical extraction of impacted 3^rd^ molars specifically for pain, ecchymosis, and edema. Further randomized clinical trials with a larger group of subjects are warranted to confirm these findings.

## Figures and Tables

**Table 1:** Demographics of included patients.

	AM group (*n* = 22 sites)	Control group (*n* = 8 sites)
Age (mean/years)	26	24
Gender	15 females/7 males	8 females
Classification of impacted mandibular 3^rd^ molar		
Depth B	12 (54.5%)	4 (50.0%)
Depth C	10 (45.5%)	4 (50.0%)
Class II	11 (50.0%)	4 (50.0%)
Class III	11 (50.0%)	4 (50.0%)

**Table 2:** Collected assessment parameters by phone call from all study subjects at Days 2 and 4.

	Day 2			Day 4		
	AM group (*n* = 22)	Control group (*n* = 8)	*p* values	AM group (*n* = 22)	Control group (*n* = 8)	*p* values
Bleeding						
None	16 (72.7%)	1 (12.5%)	0.004^*∗*^	22 (100%)	8 (100%)	NA
Grade 1	4 (18.2%)	6 (75.0%)		0	0	
Grade 2	2 (9.1%)	1 (12.5%)		0	0	
Grade 3	0	0		0	0	
Swelling (edema)						
None	6 (27.3%)	1 (12.5%)	0.085	7 (31.8%)	1 (12.5%)	0.027^*∗*^
Mild	12 (54.5%)	2 (25.0%)		12 (54.5%)	2 (25.0%)	
Moderate	3 (13.6%)	5 (62.5%)		2 (9.1%)	1 (12.5%)	
Severe	1 (4.5%)	0		1 (4.5%)	4 (50.0%)	
Extraoral bruising						
None	20 (90.9%)	4 (50.0%)	0.029^*∗*^	16 (72.2%)	5 (62.5%)	0.526
Mild	2 (9.1%)	4 (50.0%)		3 (13.6%)	1 (12.5%)	
Moderate	0	0		3 (13.6%)	1 (12.5%)	
Severe	0	0		0	1 (12.5%)	
Limited mouth opening						
None	5 (22.7%)	1 (12.5%)	0.016^*∗*^	7 (31.8%)	1 (12.5%)	0.123
Mild	12 (54.5%)	2 (25.0%)		11 (50.0%)	2 (25.0%)	
Moderate	4 (18.2%)	5 (62.5%)		3 (13.6%)	3 (37.5%)	
Severe	1 (4.5%)	0		1 (4.5%)	2 (25.0%)	

^*∗*^Statistically significant.

**Table 3:** Collected assessment parameters by clinical examination from all study subjects at baseline and Day 7.

	Baseline			Day 7		
	AM group (*n* = 22)	Control group (*n* = 8)	*p* values	AM group (*n* = 22)	Control group (*n* = 8)	*p* values
Maximum mouth opening	4.79 ± 0.44	4.74 ± 0.44	0.872	4.04 ± 1.01	4.10 ± 0.54	0.565
Distance A	5.28 ± 0.93	5.18 ± 0.84	0.662	5.39 ± 0.72	5.40 ± 0.81	1.00
Distance B	8.99 ± 0.72	9.25 ± 0.69	0.662	9.12 ± 0.77	9.60 ± 0.67	0.156
Distance C	9.51 ± 0.97	9.71 ± 0.57	0.696	9.73 ± 1.01	9.98 ± 0.58	0.730
Distance D	7.87 ± 0.74	7.74 ± 0.91	0.475	8.07 ± 0.76	8.01 ± 0.87	0.730
Distance E	9.81 ± 0.57	9.58 ± 0.71	0.420	10.04 ± 0.66	9.76 ± 0.84	0.662

## Data Availability

The data supporting the conclusions of the study can be provided upon request to the corresponding author by emailing.
